# The Balance of Neutrophil Extracellular Trap Formation and Nuclease Degradation: an Unknown Role of Bacterial Coinfections in COVID-19 Patients?

**DOI:** 10.1128/mBio.03304-20

**Published:** 2021-02-16

**Authors:** Nicole de Buhr, Maren von Köckritz-Blickwede

**Affiliations:** a Department of Biochemistry, University of Veterinary Medicine Hannover, Foundation, Hannover, Germany; b Research Center for Emerging Infections and Zoonoses (RIZ), University of Veterinary Medicine Hannover, Foundation, Hannover, Germany; University of Nebraska Medical Center

**Keywords:** COVID-19, NETs, neutrophils

## Abstract

Severe acute respiratory syndrome coronavirus 2 (SARS-CoV-2) is leading to public health crises worldwide. An understanding of the pathogenesis and the development of treatment strategies is of high interest. Recently, neutrophil extracellular traps (NETs) have been identified as a potential driver of severe SARS-CoV-2 infections in humans. NETs are extracellular DNA fibers released by neutrophils after contact with various stimuli and accumulate antimicrobial substances or host defense peptides. When massively released, NETs are described to contribute to immunothrombosis in acute respiratory distress syndrome and in vascular occlusions. Based on the increasing evidence that NETs contribute to severe COVID-19 cases, DNase treatment of COVID-19 patients to degrade NETs is widely discussed as a potential therapeutic strategy. Here, we discuss potential detrimental effects of NETs and their nuclease degradation, since NET fragments can boost certain bacterial coinfections and thereby increase the severity of the disease.

## PERSPECTIVE

## NETs AND COVID-19

The increasing severity of coronavirus disease 2019 (COVID-19) worldwide has caused an immense pressure on the health care system and the scientific community to find new intervention strategies. Infection with severe acute respiratory syndrome coronavirus 2 (SARS-CoV-2) has proven to initiate an exacerbated host response in patients with severe COVID-19, which involves the massive infiltration of dysfunctional mature neutrophils into the lung as a potential risk factor (1). Generally, infiltrating neutrophils have been shown to release neutrophil extracellular traps (NETs) as a defense mechanism against invading pathogens. During the last decades, evidence showing that NETs play a crucial role in the defense mechanisms of various vertebrates, invertebrates, and plants has accumulated (2). NETs are extracellular fibers of DNA with associated histones, granule proteins (e.g., myeloperoxidase or elastase), and cationic antimicrobial peptides (3–9). They are released by activated neutrophils and were first described as an innate immune response to entrap and kill invading bacteria (7). Increasing knowledge demonstrates that NETs are built during various infectious diseases, including viral infections with influenza A virus or human immunodeficiency virus 1 (HIV-1) (10–13). In addition to having protective antimicrobial effects, aggregated NETs are able to degrade proinflammatory cytokines and thus have been shown to resolve the inflammation in gout patients (14). However, besides having protective effects, NETs have been shown to initiate several detrimental effects directly on the host, as described for thrombosis (15, 16), autoimmune diseases (17), acute respiratory distress syndrome (ARDS) (18, 19), stroke (20, 21), and other diseases (22, 23), especially when the efficient elimination of NETs is impaired (24). Furthermore, some bacteria are able to escape from NET structures via enhanced spreading inside the body (25), and some bacteria are able to use products of degraded NETs as growth factors (26). The effect of NETs on viruses is still not completely understood. However, especially for enveloped viruses, an antiviral effect of NETs was identified (12, 27, 28). Viruses can be bound and immobilized in NETs via electrostatic interactions of the positively charged molecules (e.g., histones or cathelicidins) and can attach to the negatively charged viral envelope, as was shown, for example, for influenza A virus and HIV-1 (10–12, 27, 29). In 2016, Schönrich and Raftery reviewed the mechanisms by which NETs are produced in the context of viral infection and how this may contribute to both antiviral immunity and immunopathology (10). Direct as well as indirect ways of NET induction by viral infections via antiviral pattern recognition receptors (PRRs), soluble proNET mediators, or the platelet/neutrophil axis are known. Finally, virus-induced NETs have the ability to control the virus but also damage the host (30).

Regarding COVID-19, there is clear evidence that NETs contribute to the severity of pathogenesis by damaging lung epithelial cells (31–33). Specific NET markers, like cell-free DNA, myeloperoxidase DNA (MPO-DNA), and citrullinated histone H3 (Cit-H3), are increased in sera from patients with COVID-19 compared to levels in uninfected controls (32, 34, 35). Several studies detected NETs *in vivo* in COVID-19 patients in lung tissue, blood (31, 32, 36), and tracheal aspirate fluid (31). Additionally, Mikacenic et al. have shown that soluble NET markers are increased in the bronchoalveolar lavage fluid (BALF) and alveolar spaces of patients with ventilator-associated pneumonia (37). The first studies demonstrated mechanistic explanations of how NETs are induced and contribute to COVID-19 pathogenesis. It was demonstrated that viable SARS-CoV-2 induces NETs in human neutrophils. This NET induction depends on three pathways: (i) the ROS-dependent protein arginine deiminase 4 (PAD-4) pathway, (ii) the angiotensin-converting enzyme 2 (ACE2)–serine protease axis, and (iii) virus replication (31). However, the complete mechanism of how SARS-CoV-2 induces NETs has not been known until now.

Nevertheless, NETs are strongly discussed as a potential driver of ARDS and the associated immunothrombosis (32) of COVID-19-patients (38–40). Therefore, new treatment strategies are being discussed to inhibit or destroy NETs in severe COVID-19 patients.

## THERAPEUTIC DNase TREATMENT OF COVID-19 PATIENTS

The reasons why some patients exhibit severe symptoms in COVID-19 is not well understood, and several ideas are widely discussed, including age, gender, hormones, genetic background, or immunodeficiencies (41). In this regard, it is of interest that detrimental effects of uncontrolled NET formation have been demonstrated by several authors to play a role in certain diseases, e.g., lupus nephritis (42). It is well known that the host produces DNases to keep a balance between detrimental and beneficial effects of NETs (42). As an example, patients with DNase deficiencies are more susceptible to detrimental effects of NETs in the case of lupus nephritis (42, 43).

It seems that during severe SARS-CoV-2 infections, a balanced and immune-protective NET formation is out of control. Reasons for this may be (i) noneffective or deficient host nuclease activity, (ii) massive overwhelming induction of NET formation, or (iii) a combination of both reasons.

Several studies have focused on DNase treatments for severe COVID-19 to degrade NETs. Based on the described NET formation in COVID-19 patients, it is logical to consider dornase alfa (Pulmozyme) for the treatment of severe COVID-19 ARDS (44). Interestingly, local dornase alfa treatment was tested in a calf model after infection with bovine respiratory syncytial virus (bRSV). Infections with bRSV lead to airway obstruction, as in infections with RSV in humans, and NET formation was detected in the BALF of RSV-infected infants (45). Dornase alfa treatment reduced the NET formation in the lungs; in addition, fewer airway occlusions were detected (46). Dornase alfa is a recombinant human DNase I that is able to degrade NETs and cell-free DNA and thereby act mucolytic. It is commonly used in cystic fibrosis (CF) patients, which has led to a reduced demand for antibiotics, a reduced frequency of CF-related symptoms, and improved lung function (47–51).

Currently, there are ongoing clinical trials with dornase in COVID-19 patients that intend to define the impact of aerosolized intratracheal dornase alfa administration on the severity and progression of ARDS in COVID-19 patients (52, 53). It is speculated that dornase alfa treatment of patients might promote an improved clearance of secretions and reduce extracellular double-stranded DNA-induced hyperinflammation in alveoli, preventing further damage to the lungs. Weber et al. (54) recently reported a single-center case series where dornase alfa was administered through an in-line nebulizer system to five COVID-19 patients. Data on tolerability and responses, including longitudinal values capturing respiratory function and inflammatory status, were analyzed. Following nebulized in-line administration of dornase alfa with albuterol, the fraction of inspired oxygen requirements was reduced for all five patients. Albuterol is a bronchodilator that relaxes muscles in the airways and increases airflow to the lungs and, thus, was used to increase the delivery of dornase alfa to the alveoli. Overall, no drug-associated toxicities were identified in the five patients. The results presented in this case series suggest that dornase alfa will be well tolerated by critically ill patients with COVID-19. In an experimental study, recombinant DNase I-coated polydopamine-poly(ethylene glycol) nanoparticulates (named long-acting DNase I) were generated, and the authors hypothesized that exogenous administration of long-acting DNase I may suppress SARS-CoV-2-mediated neutrophil activities and the cytokine storm (55). However, detailed clinical trials are required to formally test the dosing, safety, and efficacy of dornase alfa in COVID-19 patients. Especially, it needs to be considered that some authors describe, on the basis of *in vitro*-observed phenomena, that the degradation product of NETs might be even more cytotoxic than the intact NETs themselves (56–58).

## COINFECTIONS AS TRIGGERS OF SEVERE COVID-19 DISEASE?

Bacteria, e.g., *Staphylococcus*, *Streptococcus*, *Haemophilus*, *Pseudomonas*, and many more, are well known to induce NETs (59). Thus, coinfecting agents may also contribute to massive NET induction and associated detrimental effects. The complex influence of NETs in primary viral infections with influenza A virus and secondary bacterial coinfection with Streptococcus pneumoniae inside the ear has already been demonstrated (60).

In this context, it has also recently been discussed if early bacterial coinfections have an undefined impact during SARS-CoV-2 infections (61, 62). The study by Kreitmann et al. (61) demonstrated a higher prevalence of bacterial coinfections than of other viral infections. The main isolated pathogens were Staphylococcus aureus, Streptococcus pneumoniae, and Haemophilus influenzae. On the other hand, a recent systematic review and meta-analysis revealed that a low proportion of COVID-19 patients have a bacterial coinfection compared to proportions in previous influenza pandemics (63). However, the commonest bacteria were Mycoplasma pneumoniae, Pseudomonas aeruginosa, and Haemophilus influenzae. It was discussed by other authors that a coinfection diagnostic is complex and that antibiotic use in COVID-19 patients is high in intensive care units (64). Therefore, the authors concluded that coinfections in COVID-19 patients need good management and treatment, as well as a characterization of the coinfecting agents. Table 1 summarizes a list of bacterial pathogens found as coinfecting agents in COVID-19 patients.

**TABLE 1:** Overview of bacteria found as coinfecting agents in COVID-19 patients and their NAD biosynthesis

Bacterial coinfection identified in COVID-19 patients	Family	Gram positive or negative	Presence of bacteria lacking *de novo* NAD biosynthesis (reference)	Reference(s)
Acinetobacter baumannii	*Moraxellaceae*	Negative	No	62, 63
*Chlamydia* spp.	Chlamydiaceae	Negative	Yes (69)	62, 63
*Enterobacter* spp.	*Enterobacteriaceae*	Negative	No	62, 63
Enterococcus faecium	*Enterococcaceae*	Positive	No	62, 63
Haemophilus influenzae	*Pasteurellaceae*	Negative	Yes (68)	61, 63
Klebsiella pneumoniae	*Enterobacteriaceae*	Negative	No	62, 63
Mycoplasma pneumoniae	*Mycoplasmataceae*	Lack of a cell wall	No	62, 63
Pseudomonas aeruginosa	*Pseudomonadaceae*	Negative	No	63
Serratia marcescens	*Yersiniaceae*	Negative	No	62, 63
Staphylococcus aureus	*Staphylococcaceae*	Positive	No	61 – 63
Streptococcus pneumoniae	*Streptococcaceae*	Positive	No	61

Coinfections have been described in other pandemics in the world (like the Spanish flu) as one reason for high numbers of deaths (65, 66), and it is very important to investigate coinfections during SARS-CoV-2 infections. Compared to 1918 to 1919, the time of the Spanish flu, nowadays antibiotic treatments are widely used in intensive care units. However, having in mind that we live in a time of increasing numbers of antibiotic-resistant bacteria, preventive antibiotic treatment of COVID-19 patients without antibiograms should be avoided.

**FIG 1:**
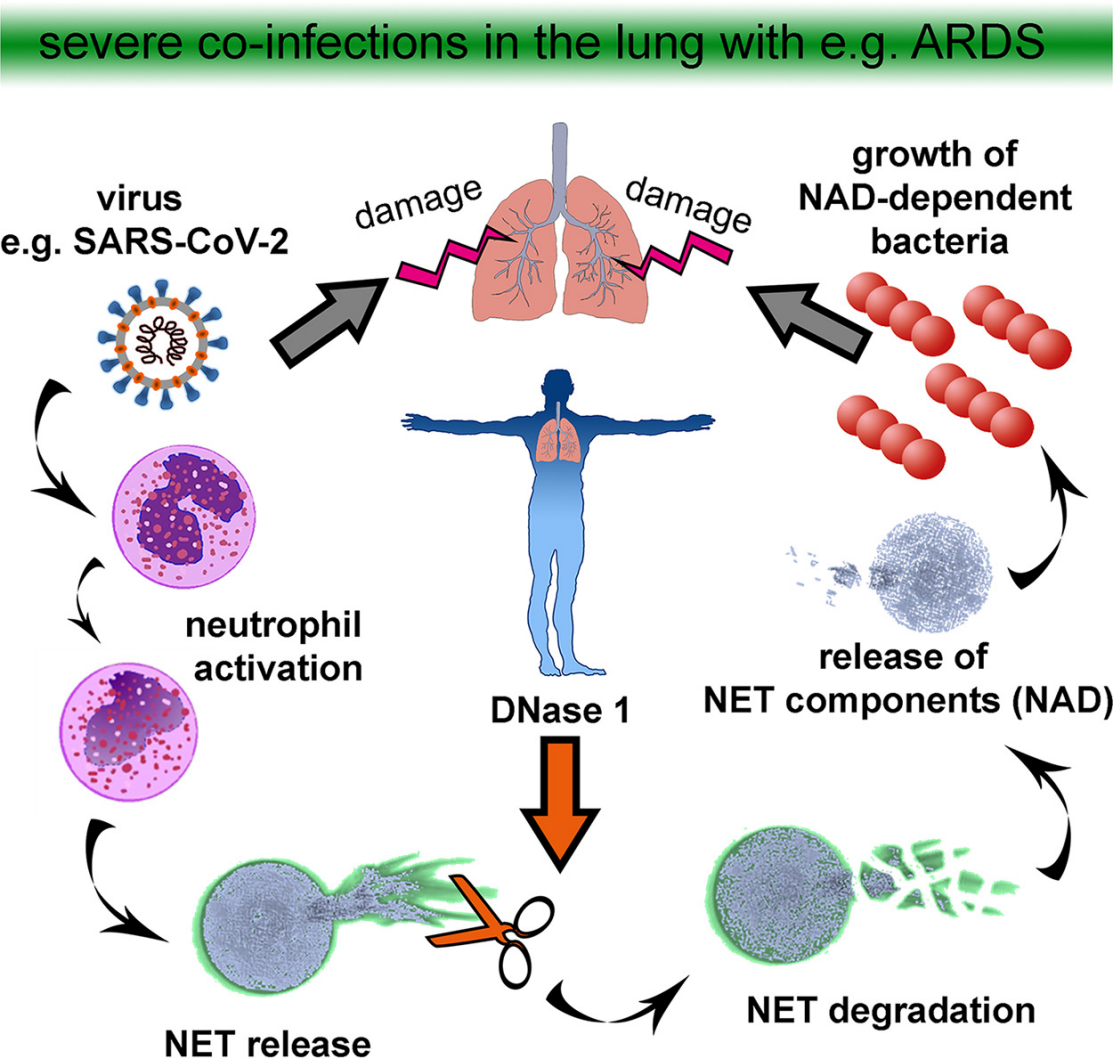
Degradation of NETs as a risk factor for severe coinfections and damage of the lung. In the case of a severe lung infection, e.g., with SARS-CoV-2, neutrophils are activated and release NETs. The host itself produces nucleases to eliminate and recycle NET products. Importantly, nuclease-mediated degradation of NETs may promote the growth of certain bacteria that use degraded NET products as an efficient source for NAD or adenosine.

In this regard, it is of high interest that we have shown in our own recent publication that nuclease-mediated degradation of NETs promotes the growth of certain bacteria (e.g., Actinobacillus pleuropneumoniae or Haemophilus influenzae) that use degraded NET products as an efficient source for NAD or adenosine (26). As Haemophilus influenzae was found in different studies as a coinfecting agent in COVID-19 patients, this phenomenon is of high interest. These bacteria can enhance their growth rate in the presence of NETs that have been efficiently degraded by the host or bacterial nucleases (DNase I and micrococcal nuclease) (Fig. 1). This effect can be diminished by inhibiting bacterial adenosine synthase, indicating that degraded NETs serve as a source for NAD.

NAD is an essential coenzyme for redox reactions and a substrate of NAD-consuming enzymes, including ADP-ribose transferases, Sir2-related protein lysine deacetylases, and bacterial DNA ligases. Therefore, targeting NAD biosynthesis in bacterial pathogens has been discussed for the development of antibacterial agents with potential broad-spectrum activity (67). However, some bacteria have evolved to depend entirely on the salvage of NAD precursors from other cells; Haemophilus influenzae and Actinobacillus pleuropneumoniae do not carry genes for a *de novo* pathway of NAD (68) and belong to the group of NAD-dependent *Pasteurellaceae*.

Interestingly, an *in vivo* infection study with Actinobacillus pleuropneumoniae demonstrated a significant influence of host DNase I inside the lung on the patho-histological severity of infected pigs (26). In pigs with a high number of lung lesions, a significantly larger amount of DNase I and a smaller amount of free DNA than in infected pigs with a low number of lung lesions were detected. These data shed light on the detrimental effects of degraded NETs during the host immune response to certain bacterial species that require and/or efficiently take advantage of degraded DNA material, which has been provided by the host nucleases. As SARS-CoV-2 does not depend on NAD and therefore does not benefit from degraded NETs, it may be hypothesized based on the findings in COVID-19 patients that underestimated bacterial infections (61) are somehow part of the severity of pathogenesis. Indeed, it may be hypothesized that an acute COVID-19 infection induces NETs and subsequently provides nutrients for sleeping Haemophilus influenzae cells, a starting point of a fatal lung infection.

Whether degraded NETs also promote the growth of other bacterial pathogens, e.g., Mycoplasma pneumoniae, Pseudomonas aeruginosa, or Chlamydiaceae, is still not known and needs further investigations, especially for coinfections with SARS-CoV-2.

## CONCLUSION: URGENT NEED FOR ADDITIONAL RESEARCH ON NETs AND COINFECTIONS IN COVID-19 PATHOGENESIS

On one hand, it is considered that a therapeutic nuclease treatment might be helpful to prevent the detrimental effects of massive NET formation during COVID-19. However, on the other hand, a nuclease treatment can impact the growth of certain NAD-dependent pathogenic bacteria, e.g., the lung pathogen Haemophilus influenzae, which can efficiently use degraded NETs as a growth factor (26). Therefore, it is necessary to include a systematic bacterial diagnostic followed by an adjusted antibiotic treatment in clinical trials with dornase alpha. As some bacteria identified in COVID-19 patients are not easy to cultivate from swap and organ samples, they may indeed be underestimated, as mentioned above. Therefore, there is an urgent need not only for additional clinical but also for experimental *in vitro* research studies focusing on bacterial coinfections in COVID-19 patients. Follow-up problems in patients may also occur with colonizing bacteria like Haemophilus influenzae if they benefit from degraded NETs and at the same time develop antibiotic resistance.

Another upcoming question is “At which time point is a DNase treatment beneficial or detrimental?” It is completely unknown if NET formation in early stages of COVID-19 may be antiviral, as enveloped viruses like SARS-CoV-2 are described to be vulnerable through NETs (10). Therefore, the effect of NETs on SARS-CoV-2 *in vitro* and *in vivo* should be investigated more in detail, as NET induction in the early phase of COVID-19 may prevent severe cases or help in specific groups, depending on age, general health status, host DNase activity, and further individual characteristics of infected people. Understanding the role of NETs in the pathogenesis of COVID-19 seems to be a key element for identifying new treatment strategies for severe and mild cases. More investigations of the complex host-pathogen interaction during SARS-CoV-2 infections are needed to clarify the influence of conceivable bacterial coinfections, NET formation, and DNase activity.

## References

[1] Wang J, Jiang M, Chen X, Montaner LJ. 2020. Cytokine storm and leukocyte changes in mild versus severe SARS-CoV-2 infection: review of 3939 COVID-19 patients in China and emerging pathogenesis and therapy concepts. J Leukoc Biol 108:17–41. doi:10.1002/JLB.3COVR0520-272R. 32534467 PMC7323250

[2] Neumann A, Brogden G, von Köckritz-Blickwede M. 2020. Extracellular traps: an ancient weapon of multiple kingdoms. Biology (Basel) 9:34. doi:10.3390/biology9020034. PMC7168307 32085405

[3] Neumann A, Völlger L, Berends ETM, Molhoek EM, Stapels DAC, Midon M, Friães A, Pingoud A, Rooijakkers SHM, Gallo RL, Mörgelin M, Nizet V, Naim HY, von Köckritz-Blickwede M. 2014. Novel role of the antimicrobial peptide LL-37 in the protection of neutrophil extracellular traps against degradation by bacterial nucleases. J Innate Immun 6:860–868. doi:10.1159/000363699. 25012862 PMC4201878

[4] de Buhr N, Reuner F, Neumann A, Stump-Guthier C, Tenenbaum T, Schroten H, Ishikawa H, Müller K, Beineke A, Hennig-Pauka I, Gutsmann T, Valentin-Weigand P, Baums CG, von Köckritz-Blickwede M. 2017. Neutrophil extracellular trap formation in the Streptococcus suis-infected cerebrospinal fluid compartment. Cell Microbiol 19:e12649. doi:10.1111/cmi.12649. 27450700

[5] Meurer M, Öhlmann S, Bonilla MC, Valentin-Weigand P, Beineke A, Hennig-Pauka I, Schwerk C, Schroten H, Baums CG, von Köckritz-Blickwede M, de Buhr N. 2020. Role of bacterial and host DNases on host-pathogen interaction during Streptococcus suis meningitis. Int J Mol Sci 21:5289. doi:10.3390/ijms21155289. PMC7432635 32722502

[6] Petretto A, Bruschi M, Pratesi F, Croia C, Candiano G, Ghiggeri G, Migliorini P. 2019. Neutrophil extracellular traps (NET) induced by different stimuli: a comparative proteomic analysis. PLoS One 14:e0218946. doi:10.1371/journal.pone.0218946. 31283757 PMC6613696

[7] Brinkmann V, Reichard U, Goosmann C, Fauler B, Uhlemann Y, Weiss D, Weinrauch Y, Zychlinsky A. 2004. Neutrophil extracellular traps kill bacteria. Science 303:1532–1535. doi:10.1126/science.1092385. 15001782

[8] Metzler KD, Fuchs TA, Nauseef WM, Reumaux D, Roesler J, Schulze I, Wahn V, Papayannopoulos V, Zychlinsky A. 2011. Myeloperoxidase is required for neutrophil extracellular trap formation: implications for innate immunity. Blood 117:953–959. doi:10.1182/blood-2010-06-290171. 20974672 PMC3035083

[9] Parker H, Albrett AM, Kettle AJ, Winterbourn CC. 2012. Myeloperoxidase associated with neutrophil extracellular traps is active and mediates bacterial killing in the presence of hydrogen peroxide. J Leukoc Biol 91:369–376. doi:10.1189/jlb.0711387. 22131345

[10] Schönrich G, Raftery MJ. 2016. Neutrophil extracellular traps go viral. Front Immunol 7:366. doi:10.3389/fimmu.2016.00366. 27698656 PMC5027205

[11] Narasaraju T, Yang E, Samy RP, Ng HH, Poh WP, Liew A-A, Phoon MC, van Rooijen N, Chow VT. 2011. Excessive neutrophils and neutrophil extracellular traps contribute to acute lung injury of influenza pneumonitis. Am J Pathol 179:199–210. doi:10.1016/j.ajpath.2011.03.013. 21703402 PMC3123873

[12] Saitoh T, Komano J, Saitoh Y, Misawa T, Takahama M, Kozaki T, Uehata T, Iwasaki H, Omori H, Yamaoka S, Yamamoto N, Akira S. 2012. Neutrophil extracellular traps mediate a host defense response to human immunodeficiency virus-1. Cell Host Microbe 12:109–116. doi:10.1016/j.chom.2012.05.015. 22817992

[13] Chan LLY, Nicholls JM, Peiris JSM, Lau YL, Chan MCW, Chan RWY. 2020. Host DNA released by NETosis in neutrophils exposed to seasonal H1N1 and highly pathogenic H5N1 influenza viruses. Respir Res 21:160. doi:10.1186/s12931-020-01425-w. 32576265 PMC7310290

[14] Schauer C, Janko C, Munoz LE, Zhao Y, Kienhöfer D, Frey B, Lell M, Manger B, Rech J, Naschberger E, Holmdahl R, Krenn V, Harrer T, Jeremic I, Bilyy R, Schett G, Hoffmann M, Herrmann M. 2014. Aggregated neutrophil extracellular traps limit inflammation by degrading cytokines and chemokines. Nat Med 20:511–517. doi:10.1038/nm.3547. 24784231

[15] Clark SR, Ma AC, Tavener SA, McDonald B, Goodarzi Z, Kelly MM, Patel KD, Chakrabarti S, McAvoy E, Sinclair GD, Keys EM, Allen-Vercoe E, DeVinney R, Doig CJ, Green FHY, Kubes P. 2007. Platelet TLR4 activates neutrophil extracellular traps to ensnare bacteria in septic blood. Nat Med 13:463–469. doi:10.1038/nm1565. 17384648

[16] Fuchs TA, Brill A, Duerschmied D, Schatzberg D, Monestier M, Myers DD, Wrobleski SK, Wakefield TW, Hartwig JH, Wagner DD. 2010. Extracellular DNA traps promote thrombosis. Proc Natl Acad Sci U S A 107:15880–15885. doi:10.1073/pnas.1005743107. 20798043 PMC2936604

[17] Pieterse E, van der Vlag J. 2014. Breaking immunological tolerance in systemic lupus erythematosus. Front Immunol 5:164. doi:10.3389/fimmu.2014.00164. 24782867 PMC3988363

[18] Liu S, Su X, Pan P, Zhang L, Hu Y, Tan H, Wu D, Liu B, Li H, Li H, Li Y, Dai M, Li Y, Hu C, Tsung A. 2016. Neutrophil extracellular traps are indirectly triggered by lipopolysaccharide and contribute to acute lung injury. Sci Rep 6:37252. doi:10.1038/srep37252. 27849031 PMC5110961

[19] Caudrillier A, Kessenbrock K, Gilliss BM, Nguyen JX, Marques MB, Monestier M, Toy P, Werb Z, Looney MR. 2012. Platelets induce neutrophil extracellular traps in transfusion-related acute lung injury. J Clin Invest 122:2661–2671. doi:10.1172/JCI61303. 22684106 PMC3386815

[20] Vallés J, Lago A, Santos MT, Latorre AM, Tembl J, Salom J, Nieves C, Moscardó A. 2017. Neutrophil extracellular traps are increased in patients with acute ischemic stroke: prognostic significance. Thromb Haemost 117:1919–1929. doi:10.1160/TH17-02-0130. 28837206

[21] Thålin C, Demers M, Blomgren B, Wong SL, von Arbin M, von Heijne A, Laska AC, Wallén H, Wagner DD, Aspberg S. 2016. NETosis promotes cancer-associated arterial microthrombosis presenting as ischemic stroke with troponin elevation. Thromb Res 139:56–64. doi:10.1016/j.thromres.2016.01.009. 26916297 PMC4769435

[22] Söderberg D, Segelmark M. 2016. Neutrophil extracellular traps in ANCA-associated vasculitis. Front Immunol 7:256. doi:10.3389/fimmu.2016.00256. 27446086 PMC4928371

[23] Meegan JE, Yang X, Coleman DC, Jannaway M, Yuan SY. 2017. Neutrophil-mediated vascular barrier injury: role of neutrophil extracellular traps. Microcirculation 24:e12352. doi:10.1111/micc.12352. PMC5404986 28120468

[24] Mayadas TN, Cullere X, Lowell CA. 2014. The multifaceted functions of neutrophils. Annu Rev Pathol 9:181–218. doi:10.1146/annurev-pathol-020712-164023. 24050624 PMC4277181

[25] Beiter K, Wartha F, Albiger B, Normark S, Zychlinsky A, Henriques-Normark B. 2006. An endonuclease allows Streptococcus pneumoniae to escape from neutrophil extracellular traps. Curr Biol 16:401–407. doi:10.1016/j.cub.2006.01.056. 16488875

[26] de Buhr N, Bonilla MC, Pfeiffer J, Akhdar S, Schwennen C, Kahl BC, Waldmann K, Valentin-Weigand P, Hennig-Pauka I, von Köckritz-Blickwede M. 2019. Degraded neutrophil extracellular traps promote the growth of Actinobacillus pleuropneumoniae. Cell Death Dis 10:657. doi:10.1038/s41419-019-1895-4. 31506432 PMC6736959

[27] Hoeksema M, Tripathi S, White M, Qi L, Taubenberger J, van Eijk M, Haagsman H, Hartshorn KL. 2015. Arginine-rich histones have strong antiviral activity for influenza A viruses. Innate Immun 21:736–745. doi:10.1177/1753425915593794. 26138524 PMC5431080

[28] Galani IE, Andreakos E. 2015. Neutrophils in viral infections: current concepts and caveats. J Leukoc Biol 98:557–564. doi:10.1189/jlb.4VMR1114-555R. 26160849

[29] Wardini AB, Guimaraes-Costa AB, Nascimento MTC, Nadaes NR, Danelli MGM, Mazur C, Benjamim CF, Saraiva EM, Pinto-da-Silva LH. 2010. Characterization of neutrophil extracellular traps in cats naturally infected with feline leukemia virus. J Gen Virol 91:259–264. doi:10.1099/vir.0.014613-0. 19793908

[30] Jenne CN, Kubes P. 2015. Virus-induced NETs—critical component of host defense or pathogenic mediator? PLoS Pathog 11:e1004546. doi:10.1371/journal.ppat.1004546. 25569217 PMC4287541

[31] Veras FP, Pontelli MC, Silva CM, Toller-Kawahisa JE, de Lima M, Nascimento DC, Schneider AH, Caetité D, Tavares LA, Paiva IM, Rosales R, Colón D, Martins R, Castro IA, Almeida GM, Lopes MIF, Benatti MN, Bonjorno LP, Giannini MC, Luppino-Assad R, Almeida SL, Vilar F, Santana R, Bollela VR, Auxiliadora-Martins M, Borges M, Miranda CH, Pazin-Filho A, da Silva LLP, Cunha LD, Zamboni DS, Dal-Pizzol F, Leiria LO, Siyuan L, Batah S, Fabro A, Mauad T, Dolhnikoff M, Duarte-Neto A, Saldiva P, Cunha TM, Alves-Filho JC, Arruda E, Louzada-Junior P, Oliveira RD, Cunha FQ. 2020. SARS-CoV-2-triggered neutrophil extracellular traps mediate COVID-19 pathology. J Exp Med 217:e20201129. doi:10.1084/jem.20201129. 32926098 PMC7488868

[32] Middleton EA, He X-Y, Denorme F, Campbell RA, Ng D, Salvatore SP, Mostyka M, Baxter-Stoltzfus A, Borczuk AC, Loda M, Cody MJ, Manne BK, Portier I, Harris ES, Petrey AC, Beswick EJ, Caulin AF, Iovino A, Abegglen LM, Weyrich AS, Rondina MT, Egeblad M, Schiffman JD, Yost CC. 2020. Neutrophil extracellular traps contribute to immunothrombosis in COVID-19 acute respiratory distress syndrome. Blood 136:1169–1179. doi:10.1182/blood.2020007008. 32597954 PMC7472714

[33] Barnes BJ, Adrover JM, Baxter-Stoltzfus A, Borczuk A, Cools-Lartigue J, Crawford JM, Daßler-Plenker J, Guerci P, Huynh C, Knight JS, Loda M, Looney MR, McAllister F, Rayes R, Renaud S, Rousseau S, Salvatore S, Schwartz RE, Spicer JD, Yost CC, Weber A, Zuo Y, Egeblad M. 2020. Targeting potential drivers of COVID-19: neutrophil extracellular traps. J Exp Med 217:e20200652. doi:10.1084/jem.20200652. 32302401 PMC7161085

[34] Zuo Y, Yalavarthi S, Shi H, Gockman K, Zuo M, Madison JA, Blair CN, Weber A, Barnes BJ, Egeblad M, Woods RJ, Kanthi Y, Knight JS. 2020. Neutrophil extracellular traps in COVID-19. JCI Insight 5:e138999. doi:10.1172/jci.insight.138999. PMC7308057 32329756

[35] Zuo Y, Yalavarthi S, Shi H, Gockman K, Zuo M, Madison JA, Blair C, Weber A, Barnes BJ, Egeblad M, Woods RJ, Kanthi Y, Knight JS. 2020. Neutrophil extracellular traps (NETs) as markers of disease severity in COVID-19. medRxiv doi:10.1101/2020.04.09.20059626. PMC7308057 32329756

[36] Radermecker C, Detrembleur N, Guiot J, Cavalier E, Henket M, D’Emal C, Vanwinge C, Cataldo D, Oury C, Delvenne P, Marichal T. 2020. Neutrophil extracellular traps infiltrate the lung airway, interstitial, and vascular compartments in severe COVID-19. J Exp Med 217:e20201012. doi:10.1084/jem.20201012. 32926097 PMC7488867

[37] Mikacenic C, Moore R, Dmyterko V, West TE, Altemeier WA, Liles WC, Lood C. 2018. Neutrophil extracellular traps (NETs) are increased in the alveolar spaces of patients with ventilator-associated pneumonia. Crit Care 22:358. doi:10.1186/s13054-018-2290-8. 30587204 PMC6307268

[38] Becker RC. 2020. COVID-19 update: Covid-19-associated coagulopathy. J Thromb Thrombolysis 50:54–67. doi:10.1007/s11239-020-02134-3. 32415579 PMC7225095

[39] Zuo Y, Zuo M, Yalavarthi S, Gockman K, Madison JA, Shi H, Woodard W, Lezak SP, Lugogo NL, Knight JS, Kanthi Y. 5 November 2020. Neutrophil extracellular traps and thrombosis in COVID-19. J Thromb Thrombolysis doi:10.1007/s11239-020-02324-z. PMC7642240 33151461

[40] Makatsariya A, Slukhanchuk E, Bitsadze V, Khizroeva J, Tretyakova M, Tsibizova V, Dobryakov A, Elalamy I, Gris JC. 2020. COVID-19, neutrophil extracellular traps and vascular complications in obstetric practice. J Perinat Med 48:985–994. doi:10.1515/jpm-2020-0280. 32739908

[41] Winkler ES, Bailey AL, Kafai NM, Nair S, McCune BT, Yu J, Fox JM, Chen RE, Earnest JT, Keeler SP, Ritter JH, Kang L-I, Dort S, Robichaud A, Head R, Holtzman MJ, Diamond MS. 2020. Publisher Correction: SARS-CoV-2 infection of human ACE2-transgenic mice causes severe lung inflammation and impaired function. Nat Immunol 21:1470–1470. doi:10.1038/s41590-020-0794-2. PMC7464056 32879513

[42] Hakkim A, Furnrohr BG, Amann K, Laube B, Abed UA, Brinkmann V, Herrmann M, Voll RE, Zychlinsky A. 2010. Impairment of neutrophil extracellular trap degradation is associated with lupus nephritis. Proc Natl Acad Sci U S A 107:9813–9818. doi:10.1073/pnas.0909927107. 20439745 PMC2906830

[43] Sonawane S, Khanolkar V, Namavari A, Chaudhary S, Gandhi S, Tibrewal S, Jassim SH, Shaheen B, Hallak J, Horner JH, Newcomb M, Sarkar J, Jain S. 2012. Ocular surface extracellular DNA and nuclease activity imbalance: a new paradigm for inflammation in dry eye disease. Invest Ophthalmol Vis Sci 53:8253–8263. doi:10.1167/iovs.12-10430. 23169882 PMC3525138

[44] Earhart AP, Holliday ZM, Hofmann HV, Schrum AG. 2020. Consideration of dornase alfa for the treatment of severe COVID-19 acute respiratory distress syndrome. New Microbes New Infect 35:100689. doi:10.1016/j.nmni.2020.100689. 32355564 PMC7192073

[45] Cortjens B, de Boer OJ, de Jong R, Antonis AF, Sabogal Piñeros YS, Lutter R, van Woensel JB, Bem RA. 2016. Neutrophil extracellular traps cause airway obstruction during respiratory syncytial virus disease. J Pathol 238:401–411. doi:10.1002/path.4660. 26468056

[46] Cortjens B, de Jong R, Bonsing JG, van Woensel JBM, Antonis AFG, Bem RA. 2018. Local dornase alfa treatment reduces NETs-induced airway obstruction during severe RSV infection. Thorax 73:578–580. doi:10.1136/thoraxjnl-2017-210289. 28780505

[47] Frederiksen B, Pressler T, Hansen A, Koch C, Høiby N. 2006. Effect of aerosolized rhDNase (Pulmozyme) on pulmonary colonization in patients with cystic fibrosis. Acta Paediatr 95:1070–1074. doi:10.1080/08035250600752466. 16938752

[48] Papayannopoulos V, Staab D, Zychlinsky A. 2011. Neutrophil elastase enhances sputum solubilization in cystic fibrosis patients receiving DNase therapy. PLoS One 6:e28526. doi:10.1371/journal.pone.0028526. 22174830 PMC3235130

[49] Martínez-Alemán SR, Campos-García L, Palma-Nicolas JP, Hernández-Bello R, González GM, Sánchez-González A. 2017. Understanding the entanglement: neutrophil extracellular traps (NETs) in cystic fibrosis. Front Cell Infect Microbiol 7:104. doi:10.3389/fcimb.2017.00104. 28428948 PMC5382324

[50] Ten Berge M, van der Wiel E, Tiddens HAWM, Merkus PJFM, Hop WCJ, de Jongste JC. 2003. DNase in stable cystic fibrosis infants: a pilot study. J Cyst Fibros 2:183–188. doi:10.1016/S1569-1993(03)00090-0. 15463871

[51] Hodson ME. 1995. Aerosolized dornase Alfa (rhDNase) for therapy of cystic fibrosis. Am J Respir Crit Care Med 151:S70–S74. doi:10.1164/ajrccm/151.3_Pt_2.S70. 7881698

[52] Okur HK, Yalcin K, Tastan C, Demir S, Yurtsever B, Karakus GS, Kancagi DD, Abanuz S, Seyis U, Zengin R, Hemsinlioglu C, Kara M, Yildiz ME, Deliceo E, Birgen N, Pelit NB, Cuhadaroglu C, Kocagoz AS, Ovali E. 2020. Preliminary report of in vitro and in vivo effectiveness of dornase alfa on SARS-CoV-2 infection. New Microbes New Infect 37:100756. doi:10.1016/j.nmni.2020.100756. 32922804 PMC7476504

[53] Desilles JP, Gregoire C, Le Cossec C, Lambert J, Mophawe O, Losser MR, Lambiotte F, Le Tacon S, Cantier M, Engrand N, Trouiller P, Pottecher J. 2020. Efficacy and safety of aerosolized intra-tracheal dornase alfa administration in patients with SARS-CoV-2-induced acute respiratory distress syndrome (ARDS): a structured summary of a study protocol for a randomised controlled trial. Trials 21:548. doi:10.1186/s13063-020-04488-8. 32560746 PMC7303591

[54] Weber AG, Chau AS, Egeblad M, Barnes BJ, Janowitz T. 2020. Nebulized in-line endotracheal dornase alfa and albuterol administered to mechanically ventilated COVID-19 patients: a case series. medRxiv doi:10.1101/2020.05.13.20087734. PMC7522910 32993479

[55] Lee YY, Park HH, Park W, Kim H, Jang JG, Hong KS, Lee J-Y, Seo HS, Na DH, Kim T-H, Bin Choy Y, Ahn JH, Lee W, Park CG. 2021. Long-acting nanoparticulate DNase-1 for effective suppression of SARS-CoV-2-mediated neutrophil activities and cytokine storm. Biomaterials 267:120389. doi:10.1016/j.biomaterials.2020.120389. 33130319 PMC7583619

[56] Marsman G, Zeerleder S, Luken BM. 2016. Extracellular histones, cell-free DNA, or nucleosomes: differences in immunostimulation. Cell Death Dis 7:e2518. doi:10.1038/cddis.2016.410. 27929534 PMC5261016

[57] Marsman G, Von Richthofen H, Bulder I, Lupu F, Hazelzet J, Luken BM, Zeerleder S. 2017. DNA and factor VII-activating protease protect against the cytotoxicity of histones. Blood Adv 1:2491–2502. doi:10.1182/bloodadvances.2017010959. 29296900 PMC5728637

[58] Saffarzadeh M, Juenemann C, Queisser MA, Lochnit G, Barreto G, Galuska SP, Lohmeyer J, Preissner KT. 2012. Neutrophil extracellular traps directly induce epithelial and endothelial cell death: a predominant role of histones. PLoS One 7:e32366. doi:10.1371/journal.pone.0032366. 22389696 PMC3289648

[59] Lu T, Kobayashi SD, Quinn MT, DeLeo FR. 2012. A NET outcome. Front Immunol 3:365. doi:10.3389/fimmu.2012.00365. 23227026 PMC3514450

[60] Short KR, von Köckritz-Blickwede M, Langereis JD, Chew KY, Job ER, Armitage CW, Hatcher B, Fujihashi K, Reading PC, Hermans PW, Wijburg OL, Diavatopoulos DA. 2014. Antibodies mediate formation of neutrophil extracellular traps in the middle ear and facilitate secondary pneumococcal otitis media. Infect Immun 82:364–370. doi:10.1128/IAI.01104-13. 24191297 PMC3911859

[61] Kreitmann L, Monard C, Dauwalder O, Simon M, Argaud L. 2020. Early bacterial co-infection in ARDS related to COVID-19. Intensive Care Med 46:1787–1789. doi:10.1007/s00134-020-06165-5. 32661615 PMC7358293

[62] Lai C-C, Wang C-Y, Hsueh P-R. 2020. Co-infections among patients with COVID-19: the need for combination therapy with non-anti-SARS-CoV-2 agents? J Microbiol Immunol Infect 53:505–512. doi:10.1016/j.jmii.2020.05.013. 32482366 PMC7245213

[63] Lansbury L, Lim B, Baskaran V, Lim WS. 2020. Co-infections in people with COVID-19: a systematic review and meta-analysis. J Infect 81:266–275. doi:10.1016/j.jinf.2020.05.046. 32473235 PMC7255350

[64] Cox MJ, Loman N, Bogaert D, O'Grady J. 2020. Co-infections: potentially lethal and unexplored in COVID-19. Lancet Microbe 1:e11. doi:10.1016/S2666-5247(20)30009-4. 32835323 PMC7195315

[65] Morens DM, Taubenberger JK, Fauci AS. 2008. Predominant role of bacterial pneumonia as a cause of death in pandemic influenza: implications for pandemic influenza preparedness. J Infect Dis 198:962–970. doi:10.1086/591708. 18710327 PMC2599911

[66] McCullers JA. 2014. The co-pathogenesis of influenza viruses with bacteria in the lung. Nat Rev Microbiol 12:252–262. doi:10.1038/nrmicro3231. 24590244

[67] Sorci L, Pan Y, Eyobo Y, Rodionova I, Huang N, Kurnasov O, Zhong S, MacKerell AD, Zhang H, Osterman AL. 2009. Targeting NAD biosynthesis in bacterial pathogens: structure-based development of inhibitors of nicotinate mononucleotide adenylyltransferase NadD. Chem Biol 16:849–861. doi:10.1016/j.chembiol.2009.07.006. 19716475 PMC2770502

[68] Gazzaniga F, Stebbins R, Chang SZ, McPeek MA, Brenner C. 2009. Microbial NAD metabolism: lessons from comparative genomics. Microbiol Mol Biol Rev 73:529–541. doi:10.1128/MMBR.00042-08. 19721089 PMC2738131

[69] Omsland A, Sixt BS, Horn M, Hackstadt T. 2014. Chlamydial metabolism revisited: interspecies metabolic variability and developmental stage-specific physiologic activities. FEMS Microbiol Rev 38:779–801. doi:10.1111/1574-6976.12059. 24484402 PMC4790414

